# Genomes of *Syntrophomonas erecta* GB4-38^T^ and *Syntrophomonas erecta* subsp. *sporosyntropha* 5-3-Z^T^ that degrade C4-8 short-chain fatty acids

**DOI:** 10.1128/mra.01419-25

**Published:** 2026-02-12

**Authors:** Sung Min Ha, Ethan Humm, Neil Q. Wofford, Jessica R. Sieber, Liudmilla Rubbi, Giorgia del Vecchio, Matteo Pellegrini, Michael J. McInerney, Robert P. Gunsalus

**Affiliations:** 1 Department of Integrative Biology and Physiology, University of California, Los Angeles, California, USA; 2 Department of Microbiology, Immunology and Molecular Genetics, University of California, Los Angeles, California, USA; 3 Department of Microbiology and Plant Biology, University of Oklahoma, Norman, Oklahoma, USA; 4 Department of Molecular, Cell and Developmental Biology, University of California, Los Angeles, California, USA; 5 UCLA DOE Institute, University of California, Los Angeles, California, USA; University of Manitoba, Winnipeg, Canada

**Keywords:** *Syntrophomonas*, genomes, syntrophs, fatty acids

## Abstract

We report the genome sequences of two syntrophic gram-negative bacteria, *Syntrophomonas erecta* GB4-38^**T**^ and *Syntrophomonas erecta* subsp. *sporosyntropha* 5-3-Z^**T**^, which degrade C4-C8 short-chain fatty acids when grown in co-culture with a suitable hydrogen-consuming microbial strain.

## ANNOUNCEMENT

Two syntrophic bacterial strains assigned to the genus *Syntrophomonas* within the family Syntrophomonadaceae grow on C4 to C8 fatty acid substrates only when co-cultured with a suitable hydrogen-consuming partner. Both partners must cooperate to allow fatty acid metabolism to be thermodynamically favorable and allow energy conservation. *Syntrophomonas erecta* GB4-38^**T**^ was isolated from granular sludge obtained from an upflow anaerobic sludge blanket reactor treating bean-curd farm wastewater (1). *Syntrophomonas erecta* subsp. *sporosyntropha* 5-3-Z^**T**^ was obtained from a river sediment inoculum (2). Both species can be routinely grown in pure culture using pre-reduced mineral media supplemented with the fermentable substrate, crotonate, as a sole carbon and energy source (3). The microbial syntrophic process of fatty acid oxidation is widely distributed in natural and manmade habitats, although the underlying genetic and biochemical basis remains poorly understood (4, 5). The availability of these newly sequenced genomes should provide insight regarding the syntrophic operations performed by the genus *Syntrophomonas*.

The *S. erecta* GB4-38^**T**^ (=DSM 16215^T^) and *S. erecta* subsp. *sporosyntropha* 5-3-Z^**T**^ (=JCM 13344^T^) strains were obtained from the McInerney laboratory culture collection and cultured anaerobically at 37°C on a crotonate mineral salts medium as previously described (3). DNA was extracted using the Zymo D6060 Quick-DNA HMW Magbead Kit (Irvine, CA, USA) per the manufacturer’s instructions and fragmented using Covaris g-Tubes following instructions from the manufacturer (4 passes at 7,000 rpm through the g-Tube orifice). The average size of the sheared gDNA was checked at the TapeStation 4200 (Agilent, Santa Clara, CA, USA). Multiplexed microbial libraries were prepared using the PacBio SMRTbell prep kit 3.0 together with the SMRTbell barcoded adapters 3.0 according to the PacBio protocol (Menlo Park, CA, USA). Final whole genome libraries were not size selected but simply purified via a standard procedure using 1× SMRTbell clean up beads. DNA sequencing was performed using the PacBio Sequel IIe platform. High-quality reads were assembled by Canu version 2.2 (6), and the assembled genomes were further refined by Circlator version 1.5.5 (7) to identify circular contigs and remove redundant non-circular contigs. A completeness check was performed by CheckM version 1.0.18 (8), and the *N*_50_ quality was determined by Assembly stats version 1.01 (https://github.com/sanger-pathogens/assembly-stats). Genome ORF calling and annotation were performed by NCBI PGAP version 6.10 (9). Default parameters were used for all tools. Properties of the *S. erecta* GB4-38^**T**^ and *S. erecta* subsp. *sporosyntropha* 5-3-Z^**T**^ genomes are shown in Table 1. Genomic 16S gene sequences were also compared to the published 16S sequence of the two described type strains (1, 2), indicating close agreement (Table 1).

**TABLE 1:** *S. erecta* GB4-38^**T**^ and *S. erecta* subsp. *sporosyntropha* 5-3-Z**^T^** genome sequence information

Strain	*S. erecta* GB4-38ᵀ	*S. erecta* subsp. *sporosyntropha* 5-3-Zᵀ
NCBI Taxonomy ID	264446	370896
Culture collection ID	DSM 16215ᵀ	JCM 13344ᵀ
Contigs	1	1
Topology	Circular	Circular
Size (bp)	2,650,366	2,736,411
GC% isolation	43.2	40.6
GC% from the genome	42.78	42.88
Protein-coding genes	2,434	2,557
16S rRNA number	3	3
tRNA number	48	48
Coverage (×)	45.94	42.18
Raw reads	212,536	39,596
High-quality reads	11,540	13,318
*N*_50_ (bp)	2,650,366	2,736,411
Completeness (%)	97.35	97.35
16S identity (%)	99.74–99.94^*a*^	99.54–100^*b*^
Isolation source	Bean-curd farm wastewater	River sediment
Location	Beijing, China	Beijing, China

^*a*^ Genomic 16S sequences to the S. *erecta* GB4-38**^T^** GenBank/EMBL/DDBJ accession no. AY536889.1 determined by NCBI BLASTN 2.17.0+ (10).

^*b*^ Genomic 16S sequences to the *S. erecta* subsp. *sporosyntropha* 5-3-Z**^T^** GenBank/EMBL/DDBJ accession no. DQ086234.1 determined by NCBI BLASTN 2.17.0+ (10).

The genomes of both *Syntrophomonas* strains possess multiple gene clusters encoding hydrogenase and formate dehydrogenase enzymes plus genes for EMO, a membrane-bound oxidoreductase, and electron transfer factor proteins involved in reverse electron transfer reactions (5, 11, 12). Both strains also have multiple paralogs for beta-oxidation pathway enzymes to metabolize short-chain fatty acids (4, 5).

The genome sequences will provide insight into the mechanisms for anaerobic compound degradation in the absence of added electron acceptors and will guide future studies on this class of microbes.

## Data Availability

The *S. erecta* GB4-38^T^ raw sequencing reads have been deposited under accession number SRR35925417 in the NCBI BioProject PRJNA1333567, and the assembled genome is listed under the GenBank accession number GCA_052919835.1. The *S. erecta* subsp. *sporosyntropha* 5-3-Z^T^ raw sequencing reads have been deposited under accession number SRR35928053 in the NCBI BioProject PRJNA1334587, and the assembled genome is listed under the GenBank accession number GCA_052976805.1. The *S. erecta* GB4-38^T^ IMG genome OID number is 8030286552 while the S. erecta subsp. sporosyntropha 5-3-Z^T^ IMG genome OID number is 8025877871.

## References

[1] Zhang C, Liu X, Dong X. 2005. Syntrophomonas erecta sp. nov., a novel anaerobe that syntrophically degrades short-chain fatty acids. Int J Syst Evol Microbiol 55:799–803. doi:10.1099/ijs.0.63372-0 15774665

[2] Wu C, Liu X, Dong X. 2006. Syntrophomonas erecta subsp. sporosyntropha subsp. nov., a spore-forming bacterium that degrades short chain fatty acids in co-culture with methanogens. Syst Appl Microbiol 29:457–462. doi:10.1016/j.syapm.2006.01.003 16455220

[3] McInerney M.J, Bryant MP, Pfennig N. 1979. Anaerobic bacterium that degrades fatty acids in syntrophic association with methanogens. Arch Microbiol 122:129–135. doi:10.1007/BF00411351 9914307

[4] McInerney MJ, Struchtemeyer CG, Sieber J, Mouttaki H, Stams AJM, Schink B, Rohlin L, Gunsalus RP. 2008. Physiology, ecology, phylogeny, and genomics of microorganisms capable of syntrophic metabolism. Ann N Y Acad Sci 1125:58–72. doi:10.1196/annals.1419.005 18378587

[5] Sieber JR, McInerney MJ, Gunsalus RP. 2012. Genomic insights into syntrophy: the paradigm for anaerobic metabolic cooperation. Annu Rev Microbiol 66:429–452. doi:10.1146/annurev-micro-090110-102844 22803797

[6] Koren S, Walenz BP, Berlin K, Miller JR, Bergman NH, Phillippy AM. 2017. Canu: scalable and accurate long-read assembly via adaptive k-mer weighting and repeat separation. Genome Res 27:722–736. doi:10.1101/gr.215087.116 28298431 PMC5411767

[7] Hunt M, Silva ND, Otto TD, Parkhill J, Keane JA, Harris SR. 2015. Circlator: automated circularization of genome assemblies using long sequencing reads. Genome Biol 16:294. doi:10.1186/s13059-015-0849-0 26714481 PMC4699355

[8] Parks DH, Imelfort M, Skennerton CT, Hugenholtz P, Tyson GW. 2015. CheckM: assessing the quality of microbial genomes recovered from isolates, single cells, and metagenomes. Genome Res 25:1043–1055. doi:10.1101/gr.186072.114 25977477 PMC4484387

[9] Li W, O’Neill KR, Haft DH, DiCuccio M, Chetvernin V, Badretdin A, Coulouris G, Chitsaz F, Derbyshire MK, Durkin AS, Gonzales NR, Gwadz M, Lanczycki CJ, Song JS, Thanki N, Wang J, Yamashita RA, Yang M, Zheng C, Marchler-Bauer A, Thibaud-Nissen F. 2021. RefSeq: expanding the prokaryotic genome annotation pipeline reach with protein family model curation. Nucleic Acids Res 49:D1020–D1028. doi:10.1093/nar/gkaa1105 33270901 PMC7779008

[10] Zhang Z, Schwartz S, Wagner L, Miller W. 2000. A greedy algorithm for aligning DNA sequences. J Comput Biol 7:203–214. doi:10.1089/10665270050081478 10890397

[11] Sieber JR, Sims DR, Han C, Kim E, Lykidis A, Lapidus AL, McDonnald E, Rohlin L, Culley DE, Gunsalus R, McInerney MJ. 2010. The genome of Syntrophomonas wolfei: new insights into syntrophic metabolism and biohydrogen production. Environ Microbiol 12:2289–2301. doi:10.1111/j.1462-2920.2010.02237.x 21966920

[12] Agne M, Estelmann S, Seelmann CS, Kung J, Wilkens D, Koch H-G, van der Does C, Albers SV, von Ballmoos C, Simon J, Boll M. 2021. The missing enzymatic link in syntrophic methane formation from fatty acids. Proc Natl Acad Sci USA 118:e2111682118. doi:10.1073/pnas.2111682118 34583996 PMC8501807

